# The potential role of astroglial GABA_A_ receptors in autoimmune encephalitis associated with GABA_A_ receptor antibodies and seizures

**DOI:** 10.1002/epi4.12750

**Published:** 2023-05-02

**Authors:** Fatme Seval Ismail, Pedro M. Faustmann

**Affiliations:** ^1^ Department of Neurology, Klinikum Vest Academic Teaching Hospital of the Ruhr University Bochum Recklinghausen Germany; ^2^ Department of Neuroanatomy and Molecular Brain Research Ruhr University Bochum Bochum Germany

**Keywords:** antibodies, astrocytes, autoimmune encephalitis, GABA_A_ receptor, seizures

## Abstract

The γ‐aminobutyric acid (GABA) is the main inhibitory transmitter in the central nervous system and GABA receptors mediate the inhibitory synaptic transmission. GABA binding to neuronal GABA_A_R leads to a rapid hyperpolarization and a higher excitation threshold due to an increase in membrane Cl^−^ permeability. The synaptic GABA_A_R is mostly composed of two α(1–3), two β, and one γ subunit with the most abundant configuration α1β2γ2. Recently, antibodies (Abs) against α1, β3, and γ2 subunits of GABA_A_R were detected in a severe form of autoimmune encephalitis with refractory seizures, status epilepticus, and multifocal brain lesions, affecting gray and white matter. Experimental studies confirmed multiple mechanisms and direct functional effects of GABA_A_R Abs on neurons with decreased GABAergic synaptic transmission and increased neuronal excitability. The expression of GABA_A_R on astrocytes is well established. However, extensive studies about the effects of autoimmune GABA_A_R Abs on astrocytic GABA_A_R are missing. We hypothesize that GABA_A_R Abs may lead additionally to blocking astrocytic GABA_A_Rs with impaired Ca^2+^ homeostasis/spreading, astrocytic Cl^−^ imbalance, dysfunction of astrocyte‐mediated gliotransmission (e.g., decreased adenosine levels) and accumulation of excitatory neurotransmission, all this contributing to seizures, variable clinical/MRI presentations, and severity. The most abundant expressed GABA_A_R subunits in rodent astrocytes are α1, α2, β1, β3, and γ1 localized in both white and gray matter. Data about GABA_A_R subunits in human astrocytes are even more limited, comprising α2, β1, and γ1. Overlapping binding of GABA_A_R Abs to neuronal and astroglial receptors is still possible. In vitro and in vivo animal models can be helpful to test the effects of GABA_A_R Abs on glia. This is from an epileptological point of view relevant because of the increasing evidence, confirming the glial involvement in the pathogenesis of epilepsy. Taken together, autoimmune disorders are complex and multiple mechanisms including glia could contribute to the pathogenesis of GABA_A_R encephalitis with seizures.

The γ‐aminobutyric acid (GABA) is the main inhibitory transmitter in the central nervous system (CNS) and GABA receptors mediate the inhibitory synaptic transmission.^1^ Three types of GABA receptors can be distinguished: the GABA_A_R and GABA_C_R, both ligand‐gated chloride (Cl^−^) channels, and the G‐protein‐coupled GABA_B_R, inhibiting neuronal activities via reduction of exocytosis/transmitter release, membrane hyperpolarization, and depolarization shunt. The neuronal GABA_B_R is coupled to K^+^ or Ca^2+^ channels by second messenger systems including G‐proteins, resulting in the inhibition of presynaptical transmitter release or in hyperpolarization of postsynaptical neurons.^2^ GABA binding to neuronal GABA_A_R leads to a rapid hyperpolarization and a higher excitation threshold, for example, an inhibitory postsynaptic potential, due to an increase in membrane Cl^−^ permeability.^2^ The GABA_A_R is composed of five subunits which form a heteropentamer from 19 isoforms (α1–6, β1–3, γ1–3, δ, ε, π, θ, and σ1–3).^3^, ^4^, ^5^ The synaptic GABA_A_R is mostly composed of two α(1–3), two β, and one γ subunit with the most abundant configuration α1β2γ2. Otherwise, the peri‐ or extrasynaptic receptors consist of α4/α6 with β and δ.^3^, ^6^, ^7^ Both phasic and tonic inhibition is mediated by GABA_A_Rs.^8^, ^9^ GABA_A_Rs mediating tonic inhibition differ from those mediating phasic inhibition with regard to receptor localization and subunit composition. GABA_A_Rs mediating phasic inhibition are synaptically localized and have a low affinity for GABA, resulting in transient, rapidly desensitizing GABAergic conductance. In contrast, tonic inhibition is mediated by extrasynaptic GABA_A_Rs, which have a high affinity for GABA, leading to persistent GABAergic conductance.^10^ It has been reported that the tonic inhibition is due to GABA released from glial cells and its permeation through the glial anion channel Bestrophin 1.^11^ Moreover, the subunit distribution is varying across brain regions and cell types.^10^, ^12^ The α1 subunit expression was found at high levels in the olfactory bulb, cortex, thalamus, cerebellum, and hippocampus.^10^ Similarly, the α2 subunit was detected at high levels in the cortex, striatum, olfactory bulb, amygdala, hippocampus, and bed nucleus of the stria terminalis. In conclusion, the α1 and α2 subunits are widely expressed with overlapping patterns, but in an inverse relationship in their densities.^10^ In contrast, the α5 subunit is highly expressed only in the hippocampus. High expression of the β1 subunit has been identified in the cortex, hippocampus, and hypothalamus. The distributions of β2 and α1 subunits are overlapping with the highest levels of expression in the olfactory bulb, cortex, hippocampus, thalamus, striatum, and cerebellum. The β3 subunit is also highly expressed in the olfactory bulb, cortex, hippocampus, striatum, and cerebellum.^10^ Moreover, the γ2 subunit is highly expressed in the olfactory bulb, hippocampus, cortex, hypothalamus, cerebellum, striatum, thalamus, bed nucleus of the stria terminalis, and amygdala.^10^ The inhibition mediated by GABA_A_Rs, especially the tonic currents, play a crucial role in vital physiological functions, for example, regulation of neuronal excitability, network oscillations, synaptic plasticity, neurogenesis, neuronal development, information processing, higher motor function, vigilance, and cognition.^8^ GABA_A_Rs are the target of different drugs, for example, barbiturates (phenobarbital, primidone) and benzodiazepines (diazepam, lorazepam, clonazepam, midazolam, and clobazam), which have different subunit specificities and bind to distinct sites on the receptor complex, differentially regulating the opening of the Cl^−^ channel.^13^ The heterogeneity of the receptor structure as well as differences in the regional and cellular distribution provide the basis for the diversity of function and pharmacology.^4^ Accordingly, various clinical presentations of GABA_A_R pathology are possible. Epilepsy and neuropsychiatric disorders including anxiety disorders, schizophrenia, depression, and substance abuse have been reported to be caused by dysfunctional GABA_A_Rs, for example, due to mutations of α1 or β3 subunits.^3^, ^14^ In human genetic studies, multiple distinct mutations in the γ2, α1, and δ subunits have been detected in patients with epilepsy.^3^, ^15^, ^16^ Deficits in the assembly, trafficking, and function of recombinant mutant receptors have been described as possible mechanisms contributing to seizure disorders.^3^, ^15^, ^16^ Recently, antibodies (Abs) against α1, β3, and γ2 subunits of GABA_A_R have been detected in a severe form of autoimmune encephalitis with refractory seizures and status epilepticus.^6^, ^17^, ^18^ The detected Abs have only bound to a subset of GABA_A_R. This raises the question of whether other patients may have Abs to other isoforms of the GABA_A_R and why only a subset of subunits is immunogenic.^19^ GABA_A_R encephalitis, a rare form of autoimmune encephalitis, occurs at any age and presents generally with characteristic clinical‐radiologic patterns including encephalopathy and refractory seizures (to anticonvulsive drugs) with multifocal brain lesions, affecting both gray and white matter.^20^ Search for neoplasm and prompt immunotherapy should be done in adults because GABA_A_R encephalitis is treatable.^20^ Another open question is whether GABA_A_R Abs themselves contribute to the brain lesions or other immune mechanisms or cells (e.g., astrocytes) are involved. According to the distinctive neuroimaging phenotype of GABA_A_R encephalitis, a positive correlation between the distribution of brain MRI lesions and gene expression level of β3 subunit–containing GABA_A_R was identified.^21^ Two clinical‐radiological types of GABA_A_R encephalitis could be distinguished based on the topology/distribution of lesions and comprise the confluent type with bilateral confluent lesions in limbic, frontal and temporal lobes, and spotted type with multiple scattered small‐to‐medium patchy lesions.^21^ The confluent type of lesions was associated with a worse prognosis and severe symptoms compared with the spotted type.^21^ From a pathophysiological point of view, on the one hand, exposure to patients’ Abs caused a reduction in the surface expression of GABA_A_R in neuronal cultures.^17^ On the other hand, experimental studies confirmed the direct functional effects of GABA_A_R Abs on the hippocampal CA1 and CA3 pyramidal neurons with decreased GABAergic synaptic transmission and increased neuronal excitability, contributing to seizure generation in GABA_A_R autoimmune encephalitis.^22^, ^23^, ^24^ Incubation with GABA_A_R Abs led to a reduced number of spontaneous inhibitory postsynaptic currents (sIPSCs) generated in CA1 pyramidal neurons but did not affect their amplitude.^22^ In pyramidal CA3 neurons, the GABA_A_R Abs decreased the number and amplitude of sIPSCs.^22^ In another study, Abs against the α1 subunit showed direct effects on the GABA_A_R function on a short time scale, diminishing GABA currents with an increase in network excitability.^25^ On longer time scales, a redistribution of the GABA_A_R localization away from synapses was found to be triggered by the Abs. Otherwise, Abs against α1γ2 subunits exerted no direct effects on GABA_A_R function.^25^ Probably, other mechanisms and actors of the immune system contribute to the Ab effects. These findings emphasize the complexity of GABA_A_R encephalitis, indicating that Abs can mediate their effect through many mechanisms within the same disease.^25^ In a further study, one Ab derived from a patient with GABA_A_R encephalitis directly competed with the neurotransmitter, changing the receptor in a resting‐like state, and the second Ab antagonized the benzodiazepine potentiation.^26^ These results confirm that mechanisms of direct functional antagonism of neurotransmission can cause GABA_A_R encephalitis in a human patient.^26^

Astrocytes are the main glia cell population in the CNS, forming complex connections with neurons, blood vessels, and other glial cells, and contributing to the maintenance of normal brain functions. Astrocytes regulate synaptic neurotransmission by being part of the tripartite synapse, which is a functional unit composed of pre‐ and postsynaptic neurons as well as perisynaptic astrocytic processes.^27^, ^28^, ^29^ Astrocytes are active participants in synaptic functions including integration, processing, and storage of synaptic information, control of synaptic transmission, and plasticity. Following this, brain function results from the coordinated activity of a network composed of both neurons and glia.^29^ Activation of metabotropic and ionotropic neurotransmitter receptors in astrocytes by neurotransmitters released from the synaptic nerve terminals can increase the intracellular Ca^2+^ level.^27^, ^28^, ^30^ The neuronal‐induced Ca^2+^ elevation in astrocytes can remain within the cell or spread to neighboring astrocytes via gap junctions, contributing to astroglial networking.^27^, ^30^ Moreover, the intracellular astrocytic Ca^2+^ increase can trigger the release of gliotransmitters such as glutamate, adenosine triphosphate (ATP), taurine, glycine, and D‐serine, which can activate pre‐ and postsynaptic receptors in neurons and regulate development and remodeling of synapses as well as the further synaptic neurotransmission.^31^, ^32^ There is an evidence that GABA acts also as a gliotransmitter.^9^ The expression of GABA_A_Rs and GABA_B_Rs on astrocytes is well established, for example, in the soma, the synapse‐surrounding processes, and the brain vessel‐contacting endfeets.^31^, ^33^, ^34^, ^35^, ^36^ Astrocytes can not only internalize GABA via receptors and transporters but also can synthesize and release GABA from internal containers and activate GABA receptors in neighboring neurons.^9^, ^31^ The most abundant GABA_A_ subunits in astrocytes are α1, α2, β1, β3, and γ1, which were partly demonstrated in both white and gray matter.^35^ The mRNAs of GABA_A_R subunits including α1–5, β1–3, γ1–3, and δ have been identified in cultured primary astrocytes from rodent.^37^, ^38^ The presence of astroglial GABA_A_Rs has been confirmed electrophysiologically, immunohistochemically, and biochemically in cultured astrocytes, brain slices, acutely isolated hippocampal slices, and membrane fractions of mature rodent brain.^34^, ^39^, ^40^ The astroglial GABA_A_Rs were demonstrated to share similar channel properties and pharmacology with the neuronal receptors.^41^, ^42^ GABA_A_Rs were expressed by astrocytes in intact tissue from rat hippocampus and showed similar pharmacological properties as previously described for cultured rat astrocytes, supporting that glial GABA_A_Rs are not an artifact of cell culture.^39^, ^43^ However, in primary cultures of rat cerebellar granule neurons and cerebellar astrocytes, the inverse benzodiazepine agonist methyl‐4‐ethyl‐6,7‐dimethoxy‐β‐carboline‐3‐carboxylate (DMCM), acting as a convulsant, reduced GABA mediated responses in neurons, but showed opposite effects on astrocytic GABA_A_Rs, resulting in enhanced GABA responses.^42^, ^44^ The different effects were explained by variations in astrocytic and neuronal GABA_A_R subunit composition, for example, due to the presence of γ1 subunit in astrocytic receptors instead of the γ2 subunit of most neuronal GABA_A_Rs.^37^ Another study demonstrated the existence of pharmacologically differing GABA_A_Rs in protoplasmic and fibrous astrocyte subtypes from rat spinal cord cultures.^44^ The local anesthetic pentobarbital and the benzodiazepine diazepam showed similar effects in both astrocyte subtypes as detected in spinal cord neurons.^44^ In contrast, DMCM led to a negative modulation of GABA responses in fibrous astrocytes, a typical response for neuronal GABA_A_Rs, whereas protoplasmic astrocytes show positive modulation by DMCM, which was attributed to the specific subunit composition of astrocytic GABA_A_Rs as mentioned above.^37^, ^44^ Interestingly, astrocytic GABA_A_Rs were reduced with in vitro aging and cerebral ischemia in activated astrocytes.^45^ Further, functional GABA_A_Rs were detected in membrane patches from the soma and processes of glial fibrillary acidic protein (GFAP)‐positive astrocytes with radial processes (so‐called radial glia‐like cells, representing astrocytic stem cells) in the adult dentate gyrus of mice, performing whole‐cell recordings and using bath application of the specific GABA_A_R agonist muscimol with subsequent reversible blocking of the responses by the GABA_A_R antagonist bicuculline.^46^ The glial GABA_A_Rs were mainly composed of the subunits α2/α4, β1, and γ1/γ3.^46^ These findings were in line with other observations with these cells.^47^

Additional evidence confirmed the existence of functional astroglial α2β1γ1 receptors in humans due to findings that astrocytes express genes for these three subunits.^48^ Activation of astroglial GABA_A_R via binding of extracellular GABA resulted in the opening of Cl^−^ channels in astrocytes from primary cell cultures and rodent hippocampal slices.^39^, ^41^ The Cl^−^‐induced depolarization caused Ca^2+^ influx from the extracellular space through voltage‐gated Ca^2+^ channels.^34^ Interestingly, in case of GABA_A_R encephalitis cortical and subcortical changes in brain MRI are described in comparison with other forms of autoimmune encephalitis.^6^ Extensive studies about additional effects of autoimmune GABA_A_R Abs on astrocytic GABA_A_R are missing.^49^ Immunocytochemical investigations from the CSF of only one patient with GABA_A_R encephalitis showed a neuronal specificity of GABA_A_R after colocalization of neuronal and glial markers.^49^ However, because of different receptor subunits and variable clinical/MRI presentations, further tissue‐based and in vitro studies are necessary for more evidence with regard to astroglial binding of GABA_A_R Abs. An astrocyte‐microglia co‐culture model of inflammation was developed by Faustmann et al. (2003), representing physiological and inflammatory conditions in the brain, and allowed to study the effects of different substances.^50^, ^51^, ^52^, ^53^, ^54^, ^55^ In the first step, it can be interesting to test the effects of GABA_A_R Abs on glia in vitro. Of course, the phenotype of glia can be affected by these culture conditions and the relevance of such experiments to better understand human pathology is limited. For this reason, neuropathologic analysis of autoimmune encephalitis cases associated with GABA_A_R Abs can be used to research underlying mechanisms similar to cases with multiple sclerosis, another autoimmune CNS disease. However, neuropathological studies in the case of GABA_A_R Abs are so far not available, because this rare disease can be identified early and patients can be treated effectively, biopsies are no longer required, and most patients survive.^18^ Therefore, in vitro and in vivo animal studies are still helpful to deepen our knowledge about pathomechanisms of human diseases, also in the case of Ab‐associated autoimmune encephalitis. We hypothesize that GABA_A_R Abs may lead additionally to dysfunction and reduction of astrocytic GABA_A_R complexes (Figure 1). Among the most abundant expressed GABA_A_R subunits in rodent astrocytes are α1 and β3, which were demonstrated in both white and gray matter,^35^ however, data about GABA_A_R subunits in human astrocytes are even more limited,^48^ so further research in this direction is necessary. The Abs in GABA_A_R encephalitis are directed against the α1 and β3 subunits, so overlapping binding to neuronal and astroglial receptors is possible. But the point about the identity of GABA_A_R subunits involved in autoimmune encephalitis compared with those expressed by astrocytes needs to be further investigated. Based on the findings of existing studies, there is a variation in the astrocytic and neuronal GABA_A_R subunit composition. The most detected GABA_A_R subunits in rodent astrocytes are α1, α2, β1, β3, and γ1 but also the mRNAs of the subunits α3–5, β2, γ2–3 and δ have been identified.^37^, ^38^ The α2, β1 and γ1 subunits at the mRNA level have been demonstrated in human astrocytes.^48^ However, Abs in autoimmune encephalitis were directed against α1, β3, and γ2 subunits of GABA_A_R, but undetected Abs to other isoforms of the GABA_A_R are possible because the distribution of the lesions and clinical symptoms vary among the patients.^6^, ^17^, ^18^

**FIGURE 1:**
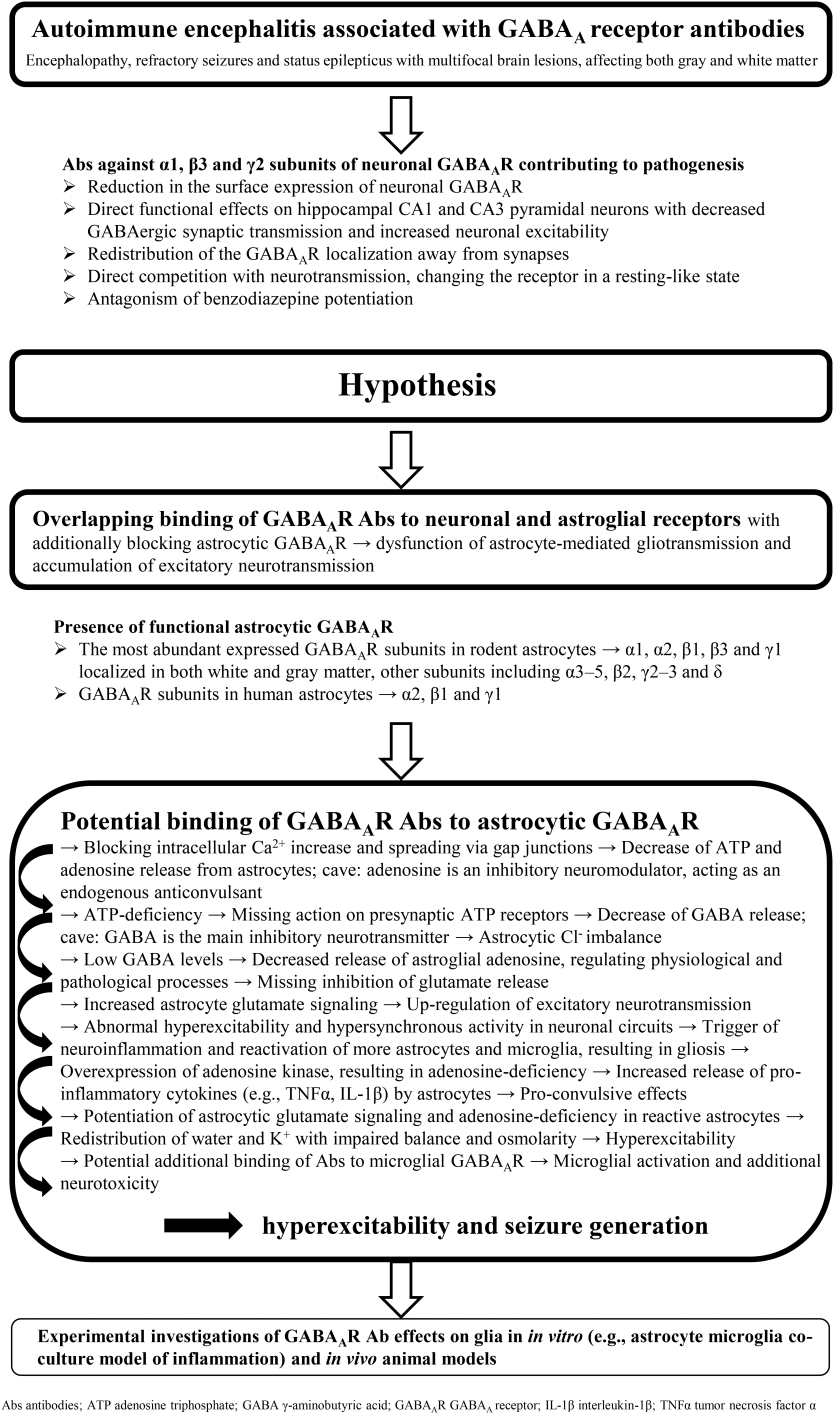
The potential role of astroglial GABA_A_ receptors in autoimmune encephalitis associated with GABA_A_ receptor antibodies and seizures. A theoretical framework of the hypothesis.

Interestingly, there is accumulating evidence of functional heterogeneity within the astrocyte population in the CNS.^56^ In the healthy, activation of GABA_A_R can increase intracellular Ca^2+^, spreading via gap junctions, and modulate the neuro‐ and gliotransmission e.g., glutamate release.^31^ The most evident route for astrocytic glutamate release is the Ca^2+^‐dependent exocytosis of vesicular glutamate from astrocytes. Previous findings showed that the glutamate released from astrocytes acts on presynaptic N‐methyl‐D‐aspartate receptor (NMDA) receptors in the hippocampal dentate gyrus and on presynaptic metabotropic glutamate receptors (mGluRs) in the hippocampal CA3‐CA1 neurons, potentiating excitatory transmission.^32^, ^56^ Astrocytic glutamate is also responsible for the synchronization of hippocampal CA1 neurons by acting on extrasynaptic NMDA receptors expressed by CA1 neurons.^57^, ^58^ Ca^2+^ and glutamate can spread via astrocytic gap junctions, composed of connexin (Cx) hexamers that form functional channels between neighboring cells, contributing to large intercellular networks. Cx43 is one of the predominant gap junction proteins in astrocytes.^59^ Moreover, Cx43‐based interglial gap junctions are also involved in seizure generation.^60^, ^61^, ^62^ During epileptogenesis, increased astrocyte glutamate signaling can contribute to excessive neuronal activity and seizures, for example, elevated Ca^2+^‐dependent glutamate gliotransmission from hyperexcitable astrocytes led to upregulation of excitatory neurotransmission in epileptic hippocampus.^63^ Besides glutamate, activation of astroglial GABA_A_R via GABA with subsequent intracellular Ca^2+^ increase also regulates the release of ATP and adenosine from astrocytes, modulating neuronal function.^31^, ^32^ Adenosine is a nucleoside and inhibitory neuromodulator, acting as an endogenous anticonvulsant. An astrocyte‐based adenosine cycle contributes to the regulation of synaptic levels of adenosine in the adult brain. Synaptic adenosine originates from extracellular degradation of ATP released by astrocytes.^64^ One of the mechanisms for astrocytic ATP release includes Cx43 and pannexin‐1 hemichannels.^64^, ^65^, ^66^ Intracellular adenosine levels are largely under the control of adenosine kinase as part of the substrate cycle between adenosine and 5′‐adenosine‐monophosphate (AMP). Moreover, astrogliosis promotes overexpression of adenosine kinase, which results in adenosine deficiency, contributing to seizure generation and aggravation.^67^, ^68^ Astroglial ATP acting on presynaptic ATP receptors can trigger a prolonged GABA release. GABA can increase the release of astroglial adenosine, which acts on presynaptic adenosine receptors to regulate physiological and pathological processes including epilepsy.^31^ In addition, ATP/adenosine released from astrocytes leads to inhibition of glutamate release from presynaptic neurons by activation of presynaptic adenosine receptors.^31^ Because of the intensive astrocyte‐neuron interactions, the astrocytic release of ATP and its subsequent degradation to adenosine plays a major regulatory role in setting global adenosine‐mediated inhibition within neuronal networks.^64^ Recently, the importance of astrocytic Cl^−^ for regulation of the excitation‐inhibition balance was demonstrated, because astrocytic Cl^−^ led to a modulation of neuronal signaling in vivo.^69^ Astrocytic GABA_A_Rs expressed in several brain regions including the cortex are often localized around inhibitory synapses with the consequence that GABA‐activating astrocytic GABA_A_Rs close to the synaptic cleft may provide Cl^−^ to maintain inhibitory transmission.^69^, ^70^ The recent data confirmed astrocytes as a dynamic reservoir for Cl^−^ (the major inhibitory anion in the CNS) that can be recruited by activation of astrocytic GABA_A_R, contributing to modulation of neuronal signaling by replenishing Cl^−^ during periods of prolonged neuronal activity. In summary, these findings support that long‐lasting inhibition is depending on astrocytic GABA_A_Rs and intracellular astrocytic [Cl^−^]_i_ because a decrease in either case can contribute to a lower threshold for seizure induction.^69^ Following this, blocking astrocytic GABA_A_R by Abs can lead to dysregulation of Ca^2+^ homeostasis and spreading via gap junctions, astrocytic Cl^−^ imbalance, dysfunction of astrocyte‐mediated gliotransmission (e.g., a decrease of adenosine levels) with the accumulation of excitatory neurotransmission. Abnormal hyperexcitability and hypersynchronous activity in neuronal circuits can trigger neuroinflammation and reactivate more astrocytes and microglia, resulting in gliosis. The reactive astrocytes undergo physicochemical and morphological changes such as hypertrophy, upregulation of many proteins, for example, metabotropic glutamate receptor (mGluR) subtypes 3, 5, and 8, the pro‐inflammatory proteins COX2 and CXCR4, nerve growth factor and its receptors, cytokines including transforming growth factor β (TGFβ), tumor necrosis factor α (TNFα), interleukins (IL) 1, 4, 6, and 10, as well as enzymes, for example, inducible nitric oxide synthase (iNOS), cathepsins D and G.^56^, ^71^, ^72^ The dynamic functions of astrocytes include the production of both pro‐ and anti‐inflammatory molecules. Hence, the release of TNFα and IL‐1β by astrocytes can cause pro‐convulsive effects depending on receptor activation and timing of expression.^73^ All these changes can lead to a potentiation of astrocytic glutamate signaling and adenosine deficiency in reactive astrocytes. Moreover, the impaired features of reactive glia to redistribute water and K^+^ with impaired balance and osmolarity could lead to hyperexcitability and seizures. Several findings suggest that both the water channel aquaporin 4 (AQP4) and the inwardly rectifying K^+^ channel (Kir4.1) are involved in the regulation of K^+^ and water levels in the brain.^56^, ^74^, ^75^ Kir4.1 is expressed in cortical astrocytes, contributing to the setting of a very negative resting potential.^74^ AQP4 is predominantly expressed in astrocytes at perivascular endfeet and regulates the water flow between the brain extracellular space and the intravascular space with maintaining the osmolarity surrounding the neurons in the brain.^75^ AQP4 is suspected to be redistributed in epileptic tissue, contributing to impaired water flux and K^+^ buffering. As a consequence of impaired astrocytic K^+^ buffering, the K^+^ clearance is expected to be attenuated, resulting in a lower seizure threshold and seizure generation.^56^, ^74^ In addition, the expression of GABA_A_R on microglia promoted a neuroprotective microglial phenotype.^76^, ^77^ Therefore, the potential binding of Abs to microglial GABA_A_R can cause microglial activation and additional neurotoxicity. Taken together, these findings highlight the complexity of autoimmune disorders and support the hypothesis that multiple mechanisms including glia could contribute to the pathogenesis of autoimmune encephalitis associated with GABA_A_R Abs and seizure generation.

These considerations are from an epileptological point of view interesting because there is increasing evidence, confirming the involvement of glia in the pathogenesis of epilepsy.^78^, ^79^ Moreover, glial cells including astrocytes and microglia are potential players in future treatment strategies for epilepsy, especially as “add‐on” therapy in addition to classical anticonvulsive drugs targeting neuronal cells.^80^, ^81^ The astrocyte‐microglia co‐culture model has the potential to be used for the differentiation of novel antiepileptogenic targets.

## CONFLICT OF INTEREST STATEMENT

None of the authors has any conflict of interest to disclose.

## ETHICS STATEMENT

We confirm that we have read the Journal's position on issues involved in ethical publication and affirm that this report is consistent with those guidelines.

## References

[1] Krnjević K , Schwartz S . The action of gamma‐aminobutyric acid on cortical neurones. Exp Brain Res. 1967;3(4):320–36. 10.1007/BF00237558 6031164

[2] Bormann J . Electrophysiology of GABAA and GABAB receptor subtypes. Trends Neurosci. 1988;11(3):112–6. 10.1016/0166-2236(88)90156-7 2465608

[3] Jacob TC , Moss SJ , Jurd R . GABA(A) receptor trafficking and its role in the dynamic modulation of neuronal inhibition. Nat Rev Neurosci. 2008;9(5):331–43. 10.1038/NRN2370 18382465 PMC2709246

[4] Olsen RW , Sieghart W . GABA a receptors: subtypes provide diversity of function and pharmacology. Neuropharmacology. 2009;56(1): 141–8. 10.1016/J.NEUROPHARM.2008.07.045 18760291 PMC3525320

[5] Olsen RW , Sieghart W . International Union of Pharmacology. LXX. Subtypes of gamma‐aminobutyric acid(A) receptors: classification on the basis of subunit composition, pharmacology, and function. Update. Pharmacol Rev. 2008;60(3):243–60. 10.1124/PR.108.00505 18790874 PMC2847512

[6] Petit‐Pedrol M , Armangue T , Peng X , Bataller L , Cellucci T , Davis R , et al. Encephalitis with refractory seizures, status epilepticus, and antibodies to the GABAA receptor: a case series, characterisation of the antigen, and analysis of the effects of antibodies. Lancet Neurol. 2014;13(3):276–86. 10.1016/S1474-4422(13)70299-0 24462240 PMC4838043

[7] Pirker S , Schwarzer C , Wieselthaler A , Sieghart W , Sperk G . GABA(A) receptors: immunocytochemical distribution of 13 subunits in the adult rat brain. Neuroscience. 2000;101(4):815–50. 10.1016/S0306-4522(00)00442-5 11113332

[8] Farrant M , Nusser Z . Variations on an inhibitory theme: phasic and tonic activation of GABA(a) receptors. Nat Rev Neurosci. 2005;6(3):215–29. 10.1038/NRN1625 15738957

[9] Yoon BE , Lee CJ . GABA as a rising gliotransmitter. Front Neural Circuits. 2014;8:141. 10.3389/FNCIR.2014.00141 25565970 PMC4269106

[10] Lee V , Maguire J . The impact of tonic GABAA receptor‐mediated inhibition on neuronal excitability varies across brain region and cell type. Front Neural Circuits. 2014;8:3. 10.3389/FNCIR.2014.00003 24550784 PMC3909947

[11] Lee S , Yoon BE , Berglund K , Oh SJ , Park H , Shin HS , et al. Channel‐mediated tonic GABA release from glia. Science. 2010;330(6005):790–6. 10.1126/SCIENCE.1184334 20929730

[12] Sieghart W , Sperk G . Subunit composition, distribution and function of GABA(a) receptor subtypes. Curr Top Med Chem. 2002;2(8):795–816. 10.2174/1568026023393507 12171572

[13] Sills GJ , Rogawski MA . Mechanisms of action of currently used antiseizure drugs. Neuropharmacology. 2020;168:168. 10.1016/J.NEUROPHARM.2020.107966 32120063

[14] Rudolph U , Knoflach F . Beyond classical benzodiazepines: novel therapeutic potential of GABAA receptor subtypes. Nat Rev Drug Discov. 2011;10(9):685–97. 10.1038/NRD3502 21799515 PMC3375401

[15] Baulac S , Huberfeld G , Gourfinkel‐An I , Mitropoulou G , Beranger A , Prud'homme JF , et al. First genetic evidence of GABA(A) receptor dysfunction in epilepsy: a mutation in the Gamma2‐subunit gene. Nat Genet. 2001;28(1):46–8. 10.1038/NG0501-46 11326274

[16] Maljevic S , Krampfl K , Cobilanschi J , Tilgen N , Beyer S , Weber YG , et al. A mutation in the GABA(A) receptor alpha(1)‐subunit is associated with absence epilepsy. Ann Neurol. 2006;59(6):983–7. 10.1002/ANA.20874 16718694

[17] Pettingill P , Kramer HB , Coebergh JA , Pettingill R , Maxwell S , Nibber A , et al. Antibodies to GABAA receptor Α1 and Γ2 subunits: clinical and serologic characterization. Neurology. 2015;84(12):1233–41. 10.1212/WNL.0000000000001326 25636713 PMC4366091

[18] Spatola M , Petit‐Pedrol M , Simabukuro MM , Armangue T , Castro FJ , Artigues MIB , et al. Investigations in GABA a receptor antibody‐associated encephalitis. Neurology. 2017;88(11):1012–20. 10.1212/WNL.0000000000003713 28202703 PMC5384834

[19] Lancaster E . Encephalitis, severe seizures, and multifocal brain lesions: recognizing autoimmunity to the GABAA receptor. Neurol Neuroimmunol Neuroinflammation. 2019;6(3):554. 10.1212/NXI.0000000000000554 PMC6467683 31044145

[20] O'Connor K , Waters P , Komorowski L , Zekeridou A , Guo CY , Mgbachi VC , et al. GABAA receptor autoimmunity: a multicenter experience. Neurol Neuroimmunol Neuroinflammation. 2019;6(3):e552. 10.1212/NXI.0000000000000552 PMC6501640 31119187

[21] Deng B , Cai M , Qiu Y , Liu X , Yu H , Zhang X , et al. MRI characteristics of autoimmune encephalitis with autoantibodies to GABAA receptor: a case series. Neurol Neuroimmunol Neuroinflammation. 2022;9(3):e1158. 10.1212/NXI.0000000000001158 PMC8958939 35338092

[22] Menke AF , Ismail FS , Dornmair K , Cerina M , Meuth SG , Melzer N . GABA A receptor autoantibodies decrease GABAergic synaptic transmission in the hippocampal CA3 network. Int J Mol Sci. 2022;23(7):3707. 10.3390/IJMS23073707 35409067 PMC8998798

[23] Kreye J , Wright SK , van Casteren A , Stöffler L , Machule ML , Reincke SM , et al. Encephalitis patient‐derived monoclonal GABAA receptor antibodies cause epileptic seizures. J Exp Med. 2021;218(11):e20210012. 10.1084/JEM.20210012 34546336 PMC8480667

[24] Brändle SM , Cerina M , Weber S , Held K , Menke AF , Alcalá C , et al. Cross‐reactivity of a pathogenic autoantibody to a tumor antigen in GABAA receptor encephalitis. Proc Natl Acad Sci USA. 2021;118(9):e1916337118. 10.1073/PNAS.1916337118/-/DCSUPPLEMENTAL 33619082 PMC7936355

[25] van Casteren ACM , Ackermann F , Rahman KA , Andrzejak E , Rosenmund C , Kreye J , et al. Differential modes of action of Α1‐ and Α1γ2‐autoantibodies derived from patients with GABAAR encephalitis. eNeuro. 2022;9(6):ENEURO.0369‐22.2022. 10.1523/ENEURO.0369-22.2022 PMC9765394 36446572

[26] Noviello CM , Kreye J , Teng J , Prüss H , Hibbs RE . Structural mechanisms of GABAA receptor autoimmune encephalitis. Cell. 2022;185(14):2469–2477.e13. 10.1016/J.CELL.2022.06.025 35803245 PMC9394431

[27] Perea G , Araque A . GLIA modulates synaptic transmission. Brain Res Rev. 2010;63(1–2):93–102. 10.1016/J.BRAINRESREV.2009.10.005 19896978

[28] Araque A , Parpura V , Sanzgiri RP , Haydon PG . Tripartite synapses: glia, the unacknowledged partner. Trends Neurosci. 1999;22(5):208–15. 10.1016/S0166-2236(98)01349-6 10322493

[29] Perea G , Navarrete M , Araque A . Tripartite synapses: astrocytes process and control synaptic information. Trends Neurosci. 2009;32(8):421–31. 10.1016/J.TINS.2009.05.001 19615761

[30] Allen NJ , Eroglu C . Cell biology of astrocyte‐synapse interactions. Neuron. 2017;96(3):697–708. 10.1016/J.NEURON.2017.09.056 29096081 PMC5687890

[31] Liu J , Feng X , Wang Y , Xia X , Zheng JC . Astrocytes: GABAceptive and GABAergic cells in the brain. Front Cell Neurosci. 2022;16:892497. 10.3389/FNCEL.2022.892497 35755777 PMC9231434

[32] Araque A , Carmignoto G , Haydon PG , Oliet SHR , Robitaille R , Volterra A . Gliotransmitters travel in time and space. Neuron. 2014;81(4):728–39. 10.1016/J.NEURON.2014.02.007 24559669 PMC4107238

[33] Backus KH , Kettenmann H , Schachner M . Effect of benzodiazepines and pentobarbital on the GABA‐induced depolarization in cultured astrocytes. Glia. 1988;1(2):132–40. 10.1002/GLIA.440010205 2852170

[34] Fraser DD , Duffy S , Angelides KJ , Perez‐Velazquez JL , Kettenmann H , MacVicar BA . GABAA/benzodiazepine receptors in acutely isolated hippocampal astrocytes. J Neurosci. 1995;15(4):2720–32. 10.1523/JNEUROSCI.15-04-02720.1995 7722625 PMC6577765

[35] Fraser DD , Mudrick‐Donnon LA , Macvicar BA . Astrocytic GABA receptors. Glia. 1994;11(2):83–93. 10.1002/GLIA.440110203 7927650

[36] Mederos S , Perea G . GABAergic‐astrocyte signaling: a refinement of inhibitory brain networks. Glia. 2019;67(10):1842–51. 10.1002/GLIA.23644 31145508 PMC6772151

[37] Bovolin P , Santi MR , Puia G , Costa E , Grayson D . Expression patterns of gamma‐aminobutyric acid type a receptor subunit MRNAs in primary cultures of granule neurons and astrocytes from neonatal rat cerebella. Proc Natl Acad Sci USA. 1992;89(19):9344–8. 10.1073/PNAS.89.19.9344 1384051 PMC50123

[38] Zheng T , Santi MR , Bovolin P , Marlier LNJL , Grayson DR . Developmental expression of the alpha 6 GABAA receptor subunit MRNA occurs only after cerebellar granule cell migration. Brain Res Dev Brain Res. 1993;75(1):91–103. 10.1016/0165-3806(93)90068-L 8222213

[39] MacVicar BA , Tse FWY , Crichton SA , Kettenmann H . GABA‐activated Cl‐ channels in astrocytes of hippocampal slices. J Neurosci. 1989;9(10):3577–83. 10.1523/JNEUROSCI.09-10-03577.1989 2477511 PMC6569885

[40] Bureau M , Laschet J , Bureau‐Heeren M , Hennuy B , Minet A , Wins P , et al. Astroglial cells express large amounts of GABAA receptor proteins in mature brain. J Neurochem. 1995;65(5):2006–15. 10.1046/J.1471-4159.1995.65052006.X 7595484

[41] Kettenmann H , Backus KH , Schachner M . Gamma‐aminobutyric acid opens Cl‐channels in cultured astrocytes. Brain Res. 1987;404(1–2):1–9. 10.1016/0006-8993(87)91349-7 2436707

[42] Bormann J , Kettenmann H . Patch‐clamp study of gamma‐aminobutyric acid receptor Cl‐ channels in cultured astrocytes. Proc Natl Acad Sci USA. 1988;85(23):9336–40. 10.1073/PNAS.85.23.9336 2461568 PMC282734

[43] Blankenfeld G , Kettenmann H . Glutamate and GABA receptors in vertebrate glial cells. Mol Neurobiol. 1991;5(1):31–43. 10.1007/BF02935611 1687351

[44] Rosewater K , Sontheimer H . Fibrous and protoplasmic astrocytes express GABAA receptors that differ in benzodiazepine pharmacology. Brain Res. 1994;636(1):73–80. 10.1016/0006-8993(94)90177-5 8156413

[45] Tateishi N , Shimoda T , Manako J , Katsumata S , Shinagawa R , Ohno H . Relevance of astrocytic activation to reductions of astrocytic GABAA receptors. Brain Res. 2006;1089(1):79–91. 10.1016/J.BRAINRES.2006.02.139 16643860

[46] Renzel R , Sadek AR , Chang CH , Gray WP , Seifert G , Steinhäuser C . Polarized distribution of AMPA, but not GABAA, receptors in radial glia‐like cells of the adult dentate gyrus. Glia. 2013;61(7):1146–54. 10.1002/GLIA.22505 23633386

[47] Wang LP , Kempermann G , Kettenmann H . A subpopulation of precursor cells in the mouse dentate gyrus receives synaptic GABAergic input. Mol Cell Neurosci. 2005;29(2):181–9. 10.1016/J.MCN.2005.02.002 15911343

[48] Sequeira A , Shen K , Gottlieb A , Limon A . Human brain transcriptome analysis finds region‐ and subject‐specific expression signatures of GABAAR subunits. Commun Biol. 2019;2(1):153. 10.1038/S42003-019-0413-7 31069263 PMC6494906

[49] Nikolaus M , Kreye J , Turko P , Vida I , Knierim E , Prüss H . CSF reactivity in GABAA receptor antibody encephalitis–immunocytochemical distribution in the murine brain. Brain Res. 2019;1704:249–56. 10.1016/J.BRAINRES.2018.10.019 30347219

[50] Ismail FS , Corvace F , Faustmann PM , Faustmann TJ . Pharmacological investigations in glia culture model of inflammation. Front Cell Neurosci. 2021;15:15. 10.3389/FNCEL.2021.805755 PMC8716582 34975415

[51] Faustmann PM , Haase CG , Romberg S , Hinkerohe D , Szlachta D , Smikalla D , et al. Microglia activation influences dye coupling and Cx43 expression of the astrocytic network. Glia. 2003;42(2):101–8. 10.1002/GLIA.10141 12655594

[52] Corvace F , Faustmann TJ , Faustmann PM , Ismail FS . Anti‐inflammatory properties of Lacosamide in an astrocyte‐microglia Co‐culture model of inflammation. Eur J Pharmacol. 2022;915:915. 10.1016/J.EJPHAR.2021.174696 34902360

[53] Faustmann TJ , Corvace F , Faustmann PM , Ismail FS . Effects of lamotrigine and topiramate on glial properties in an astrocyte‐microglia Co‐culture model of inflammation. Int J Neuropsychopharmacol. 2022;25(3):185–96. 10.1093/IJNP/PYAB080 34791253 PMC8929754

[54] Ismail FS , Faustmann PM , Kümmel M‐L , Förster E , Faustmann TJ , Corvace F . Brivaracetam exhibits mild pro‐inflammatory features in an in vitro astrocyte‐microglia Co‐culture model of inflammation. Front Cell Neurosci. 2022;16:995861. 10.3389/FNCEL.2022.995861 36406753 PMC9670320

[55] Ismail FS , Faustmann TJ , Corvace F , Tsvetanova A , Moinfar Z , Faustmann PM . Ammonia induced microglia activation was associated with limited effects on connexin 43 and aquaporin 4 expression in an astrocyte‐microglia Co‐culture model. BMC Neurosci. 2021;22(1):21. 10.1186/S12868-021-00628-1 33765917 PMC7993489

[56] Wetherington J , Serrano G , Dingledine R . Astrocytes in the epileptic brain. Neuron. 2008;58(2):168–78. 10.1016/J.NEURON.2008.04.002 18439402 PMC4124883

[57] Angulo MC , Kozlov AS , Charpak S , Audinat E . Glutamate released from glial cells synchronizes neuronal activity in the hippocampus. J Neurosci. 2004;24(31):6920–7. 10.1523/JNEUROSCI.0473-04.2004 15295027 PMC6729611

[58] Fellin T , Pascual O , Gobbo S , Pozzan T , Haydon PG , Carmignoto G . Neuronal synchrony mediated by astrocytic glutamate through activation of Extrasynaptic NMDA receptors. Neuron. 2004;43(5):729–43. 10.1016/j.neuron.2004.08.011 15339653

[59] Dermietzel R , Hertzberg EL , Kessler JA , Spray DC . Gap junctions between cultured astrocytes: immunocytochemical, molecular, and electrophysiological analysis. J Neurosci. 1991;11(5):1421–32. 10.1523/JNEUROSCI.11-05-01421.1991 1851221 PMC6575331

[60] Mylvaganam S , Ramani M , Krawczyk M , Carlen PL . Roles of gap junctions, connexins, and pannexins in epilepsy. Front Physiol. 2014;5:5. 10.3389/FPHYS.2014.00172 24847276 PMC4019879

[61] Steinhäuser C , Seifert G , Bedner P . Astrocyte dysfunction in temporal lobe epilepsy: K+ channels and gap junction coupling. Glia. 2012;60(8):1192–202. 10.1002/GLIA.22313 22328245

[62] Dossi E , Vasile F , Rouach N . Human astrocytes in the diseased brain. Brain Res Bull. 2018;136:139–56. 10.1016/J.BRAINRESBULL.2017.02.001 28212850 PMC5766741

[63] Álvarez‐Ferradas C , Morales JC , Wellmann M , Nualart F , Roncagliolo M , Fuenzalida M , et al. Enhanced Astroglial Ca2+ signaling increases excitatory synaptic strength in the epileptic brain. Glia. 2015;63(9):1507–21. 10.1002/GLIA.22817 25980474

[64] Boison D , Chen JF , Fredholm BB . Adenosine signalling and function in glial cells. Cell Death Differ. 2010;17(7):1071–82. 10.1038/CDD.2009.131 19763139 PMC2885470

[65] Iglesias R , Dahl G , Qiu F , Spray DC , Scemes E . Pannexin 1: the molecular substrate of astrocyte "hemichannels". J Neurosci. 2009;29(21):7092–7. 10.1523/JNEUROSCI.6062-08.2009 19474335 PMC2733788

[66] Kang J , Kang N , Lovatt D , Torres A , Zhao Z , Lin J , et al. Connexin 43 hemichannels are permeable to ATP. J Neurosci. 2008;28(18):4702–11. 10.1523/JNEUROSCI.5048-07.2008 18448647 PMC3638995

[67] Fedele DE , Gouder N , Güttinger M , Gabernet L , Scheurer L , Rülicke T , et al. Astrogliosis in epilepsy leads to overexpression of adenosine kinase, resulting in seizure aggravation. Brain. 2005;128(Pt 10):2383–95. 10.1093/BRAIN/AWH555 15930047

[68] Aronica E , Zurolo E , Iyer A , De Groot M , Anink J , Carbonell C , et al. Upregulation of adenosine kinase in astrocytes in experimental and human temporal lobe epilepsy. Epilepsia. 2011;52(9):1645–55. 10.1111/J.1528-1167.2011.03115.X 21635241 PMC3169746

[69] Untiet V , Beinlich FRM , Kusk P , Kang N , Ladrón‐de‐Guevara A , Song W , et al. Astrocytic chloride is brain state dependent and modulates inhibitory neurotransmission in mice. Nat Commun. 2023;14(1):1871. 10.1038/S41467-023-37433-9 37015909 PMC10073105

[70] Riquelme R , Miralles CP , De Blas AL . Bergmann glia GABA(A) receptors concentrate on the glial processes that wrap inhibitory synapses. J Neurosci. 2002;22(24):10720–30. 10.1523/JNEUROSCI.22-24-10720.2002 12486165 PMC6758425

[71] Sofroniew MV . Astrogliosis. Cold Spring Harb Perspect Biol. 2014;7:2. 10.1101/CSHPERSPECT.A020420 PMC4315924 25380660

[72] Vezzani A , French J , Bartfai T , Baram TZ . The role of inflammation in epilepsy. Nat Rev Neurol. 2011;7(1):31–40. 10.1038/NRNEUROL.2010.178 21135885 PMC3378051

[73] Vezzani A , Granata T , Vezzani A , Granata T . Brain inflammation in epilepsy: experimental and clinical evidence. Epilepsia. 2005;46(11):1724–43. 10.1111/J.1528-1167.2005.00298.X 16302852

[74] Kucheryavykh YV , Kucheryavykh LY , Nichols CG , Maldonado HM , Baksi K , Reichenbach A , et al. Downregulation of Kir4.1 inward rectifying potassium channel subunits by RNAi impairs potassium transfer and glutamate uptake by cultured cortical astrocytes. Glia. 2007;55(3):274–81. 10.1002/GLIA.20455 17091490

[75] Nielsen S , Nagelhus EA , Amiry‐Moghaddam M , Bourque C , Agre P , Ottersen OR . Specialized membrane domains for water transport in glial cells: high‐resolution immunogold cytochemistry of Aquaporin‐4 in rat brain. J Neurosci. 1997;17(1):171–80. 10.1523/JNEUROSCI.17-01-00171.1997 8987746 PMC6793699

[76] Mead EL , Mosley A , Eaton S , Dobson L , Heales SJ , Pocock JM . Microglial neurotransmitter receptors trigger superoxide production in microglia, consequences for microglial‐neuronal interactions. J Neurochem. 2012;121(2):287–301. 10.1111/J.1471-4159.2012.07659.X 22243365

[77] Liu H , Leak RK , Hu X . Neurotransmitter receptors on microglia. Stroke Vasc Neurol. 2016;1(2):52–8. 10.1136/SVN-2016-000012 28959464 PMC5435193

[78] Patel DC , Tewari BP , Chaunsali L , Sontheimer H . Neuron‐glia interactions in the pathophysiology of epilepsy. Nat Rev Neurosci. 2019;20(5):282–97. 10.1038/S41583-019-0126-4 30792501 PMC8558781

[79] Jabs R , Seifert G , Steinhäuser C . Astrocytic function and its alteration in the epileptic brain. Epilepsia. 2008;49(suppl. 2):3–12. 10.1111/J.1528-1167.2008.01488.X 18226167

[80] Srivastava PK , Eyll J , van Godard P , Mazzuferi M , Delahaye‐Duriez A , Van Steenwinckel J , et al. A systems‐level framework for drug discovery identifies Csf1R as an anti‐epileptic drug target. Nat Commun. 2018;9(1):3561. 10.1038/S41467-018-06008-4 30177815 PMC6120885

[81] Vezzani A . Anti‐inflammatory drugs in epilepsy: does it impact Epileptogenesis? Expert Opin Drug Saf. 2015;14(4):583–92. 10.1517/14740338.2015.1010508 25645535

